# Can artificial intelligence read our handwriting? A comparison of humans and artificial intelligence in the future of assessment

**DOI:** 10.1371/journal.pone.0358694

**Published:** 2026-09-21

**Authors:** İsmail Yaşartürk, Halil İbrahim Öksüz, Ramazan Deniz, Mustafa Öztürk

**Affiliations:** Department of Primary Education, Gazi Faculty of Education, Gazi University, Ankara, Türkiye; Dr Mahalingam College of Engineering and Technology, INDIA

## Abstract

The purpose of this study is to evaluate the legibility of fourth-grade elementary school students’ handwriting and to examine the differences and relationships between two evaluators: a human researcher and artificial intelligence (ChatGPT). To this end, the study was conducted using the descriptive survey model, one of the quantitative research approaches. The study sample consisted of 67 fourth-grade students attending a public elementary school. The students’ handwriting was evaluated using the Multidimensional Legibility Scale. Writing samples were collected from each student, including copying samples, dictated samples, and free writing samples. One class period was allocated for each writing task. The 201 handwriting samples obtained from the 67 students were evaluated by a researcher (the second author) and artificial intelligence (ChatGPT). According to the findings, no significant differences were detected between the artificial intelligence and the researcher in any writing type or subdimension, except for the slant subdimension of free writing. However, a high level of positive and significant correlation was identified between the two raters. Findings indicate that artificial intelligence can produce results comparable to those of expert raters when evaluating handwriting legibility. The fact that differences among raters are not statistically significant suggests that AI-based handwriting evaluations could serve as an objective and reliable alternative.

## 1. Introduction

The assessment of writing in education reflects a student’s cognitive development and learning processes. It also plays a vital role in understanding the processes of learning to write. However, conducting standardized, objective assessments in large classrooms—and particularly during written exams—is quite challenging for teachers. Furthermore, it entails a significant workload and time commitment [1]. In this context, the assessment of writing by Artificial Intelligence (ChatGPT) demonstrates the potential to reduce teachers’ workload while offering speed, consistency, and objectivity [2,3]. With advancements in processing and deep learning models, AI has now gained the ability to analyze the unique structural characteristics of writing and score it according to evaluation criteria [4,5]. These advancements could bring a significant innovation to learning processes by evaluating not only text written in a computer environment but also in-class texts written in students’ handwriting within specific criteria. Therefore, this study focuses on the level of accuracy and effectiveness that artificial intelligence systems can achieve in detecting, understanding, and evaluating the legibility of handwritten texts at the elementary school level, compared to human evaluators.

Writing, which reflects not only students’ academic achievement but also their cognitive processes and language development, is a holistic area of learning. Furthermore, this skill is a complex process in terms of both production and evaluation, as it requires a wide range of skills, including syntax, vocabulary, metacognitive regulation, and text editing [6,7]. The skills emerging from this complex mental process must be systematically assessed [8]. Without such assessment, it becomes difficult to identify students’ strengths and weaknesses, track their progress, and adjust their learning processes based on evidence [9]. Properly assessing writing skills in the early years of elementary school plays a critical role in identifying difficulties students face or may face in a timely manner and intervening before they become permanent. Indeed, if issues encountered in the early stages of writing are not addressed, they are highly likely to carry over into later grades [10]. Furthermore, the assessment of written work is not limited to determining performance levels; it also provides important indicators of students’ thinking processes, thereby enabling an understanding of and improvement in the quality of learning [1,11]. Therefore, teachers’ regular assessment of students’ writing, identification of their weaknesses, and guidance of the writing development process are fundamental requirements for effective instruction. For this reason, writing legibility is particularly recognized as one of the most important indicators of the success of in-class instruction in elementary school [8,9]. Additionally, writing serves an educational function by revealing students’ achievements and thinking processes in elementary school. Analyzing students’ writing provides insights into the level of complex thinking they possess, and these analyses contribute to deepening learning processes [1,11]. In all these respects, writing is not merely a language skill but a fundamental pedagogical tool for monitoring, analyzing, guiding, and supporting students’ academic development.

### 1.1. Artificial intelligence in education

The use of artificial intelligence in education is not limited to course content or skill areas. It is also utilized for its multifaceted functions, such as instructional design, organizing learning processes, and making educational decisions. Chassignol and colleagues [12] note that artificial intelligence in education fulfills three core functions: administrative functions, instructional and teaching functions, and learning functions. Within the administrative function the focus of this study artificial intelligence is used in assessment processes such as checking, grading, and providing feedback on students’ written assignments. Additionally, in conjunction with smart and adaptive instructional systems, artificial intelligence facilitates the personalization of instructional processes by taking into account students’ individual learning paces, prior knowledge, and performance patterns [13]. Thanks to these AI-supported systems, students’ difficulties are identified at an early stage, while simultaneously providing teachers with evidence-based feedback to help them adjust their teaching strategies [14]. In addition, artificial intelligence can process and interpret large-scale educational data to provide detailed insights for teachers and educational administrators. This enables systematic analysis of educational gaps and students’ strengths and weaknesses, thereby increasing the potential for timely, planned interventions [15]. By streamlining time-intensive and focus-demanding tasks such as content creation, question generation, and instructional material preparation, it ensures that this time can be redirected toward pedagogical planning [16]. Indeed, it has been reported that AI-supported applications enhance student motivation and learning continuity through adaptability, personalized learning opportunities, and feedback [17]. In this context, AI should not be perceived as an element that can replace the teacher. Artificial intelligence is a complementary tool that supports learning and teaching processes and the sustainability of education. This framework establishes a foundation focused on how artificial intelligence can be structured in various areas of education (e.g., writing assessment).

### 1.2. Artificial intelligence and the assessment of writing

The critical role that the assessment of writing legibility plays in educational processes raises the question of how this process can be made more accurate, effective, consistent, and sustainable. The workload teachers face in assessing, grading, and providing feedback on writing legibility limits the time available for teaching writing [18,19]. In this context, artificial intelligence (AI) applications, which have become increasingly widespread in the field of education in recent years, are being used as a tool to support the evaluation intensity in educational processes through human-like reasoning skills. Studies supporting this view highlight the need to emphasize AI’s potential in education to analyze students’ work, score it according to predetermined criteria, and provide detailed feedback [20,21]. Additionally, Atasoy and Moslemi Nezhad Arani [20] state that generative AI tools such as ChatGPT contribute to making the assessment process more systematic and time-efficient by evaluating written texts according to predefined standards, generating scores, and supporting these scores with explanatory justifications. Such applications alleviate the assessment burden on teachers, thereby enabling them to focus on higher-order skills such as critical thinking, creativity, and argumentation in writing instruction [16,22]. Therefore, the role of artificial intelligence in assessing the readability of students’ writing is not merely a component of the instructional process but rather a managerial tool that supports assessment processes and strengthens educational decision-making.

### 1.3. The present study

Writing assessment consists of a complex series of processes that require the simultaneous evaluation of multiple dimensions, such as text structure, grammatical accuracy, contextual appropriateness, readability, and consistency [6,7]. Previous studies have shown that teachers face a significant workload when grading written assignments, and that this limits the time available for lesson planning and providing feedback [18,19]. Especially in situations requiring written assessment, given the time teachers already spend on evaluation, it is inevitable that very little time remains for feedback. There is a growing need for tools or systems that can streamline these time-consuming processes, thereby freeing up time for teachers to engage in active academic interactions with students. In other words, the need for supportive tools such as artificial intelligence in assessment processes is growing steadily. In recent years, AI systems based on language models have been proposed and adopted as supportive tools in educational processes. Consequently, there is a growing need to assess the reliability of the information provided by AI systems such as ChatGPT. Research indicates that artificial intelligence software can evaluate text written by humans according to predetermined criteria and assign appropriate scores. It is also noted that such software is capable of providing explanatory feedback regarding these scores [20]. Within the general classification of artificial intelligence applications, such uses are primarily categorized under administrative functions. This function provides teachers with support during stages—such as assessment, monitoring, and feedback—that involve a significant labor and time burden, while leaving the responsibility for pedagogical decision-making to the teachers [12]. Although the number of AI-supported studies in areas such as writing instruction, providing feedback, or developing language skills has increased, there remains a significant gap in research examining the extent to which AI can match human skills and comparing AI with human skills (see [23–25]). In particular, the extent to which AI-generated evaluations align with those of human evaluators, as well as the performance of AI in terms of accuracy and consistency, remains unclear. However, artificial intelligence systems offer advantages such as objectivity, consistency, scalability, and the ability to quickly analyze large amounts of data through automated evaluation. Nevertheless, it is emphasized that they have limitations when it comes to understanding context, establishing semantic relationships, and taking cultural characteristics into account [6,26,27]. In this context, the aim of this study is to examine the suitability of ChatGPT as a writing assessment tool and to directly compare the assessment results generated by this system with those of human evaluators. By examining the level of score agreement and evaluation accuracy between ChatGPT and human evaluators, the study also explores the potential of AI systems to support teachers in the writing assessment process. Therefore, this study is significant in that it expands the existing literature addressing the contribution of AI to teachers in educational processes, particularly regarding text readability. Additionally, it positions AI as a complementary tool that supports teachers in educational processes and aims to contribute to the ongoing discussions regarding the role of AI in education.

## 2. Methods

The aim of this study is to examine the difference and relationship between two raters by evaluating the legibility level of fourth-grade elementary school students’ handwriting using both a human researcher and artificial intelligence (ChatGPT). In line with this objective, the study was conducted using the descriptive survey model, one of the quantitative research approaches. Descriptive survey research aims to describe an existing situation as it is. In this study, the data source is defined within its existing conditions, and no intervention efforts are made [28].

### 2.1. Participants

The study sample consists of 67 fourth-grade students attending a public elementary school in the Central District of Tokat Province. The school, which serves children from families of moderate socioeconomic status, has eight fourth-grade classes. Following interviews with teachers, four classes were included in the study after the students volunteered and their families submitted fully signed data consent forms. Of the participants, 32 are female fourth-grade students and 35 are male fourth-grade students. None of the fourth-grade students participating in the study have any mental or physical disabilities. Additionally, there are no students diagnosed with a writing disorder. Therefore, the study participants are fourth-grade elementary school students with Turkish as their native language who are developing typically. The rationale for including fourth-grade elementary school students in the study is that writing skills, regardless of whether a specific learning disability is present, typically reach their final level by the third grade [29].

### 2.2. Data collection tool

The Multidimensional Legibility Scale was developed by Yıldız and Ateş [30] to measure the legibility of cursive handwriting. Following the update of the font used in elementary schools to upright basic script, it was adapted to upright basic script by Gök and Baş [31]. This scale consists of five criteria—slant, spacing, size, form, and line tracking—as well as three rating levels: “fully adequate (3),” “moderately adequate (2),” and “not at all adequate (1).” Cohen’s Kappa coefficient, which indicates the percentage of inter-rater agreement for the scale’s reliability, was calculated as .80.

### 2.3. Data collection

The necessary meetings were held with school administrators and teachers at the school where the research data was collected. After the school administration granted the necessary permissions, the data collection process began during the spring semester of the 2025–2026 academic year. The four classes selected for data collection were chosen because they were taught by four teachers who voluntarily agreed to participate in the study.

After obtaining the necessary permissions, meetings were held with teachers at the relevant school, and parental consent forms were distributed to the teachers to pass on to the parents (27/03/2026). A one-week period was allowed for the return of the signed parental consent forms (3/04/2026). Once the consent forms were obtained from the children’s parents, the data collection process began. The researcher first provided participants with information about the data collection process. Subsequently, data on copying, dictation, and free writing were collected for each student. Writing data was collected once a day from each class. On the first day, copying data was collected from all four sections. The data collection process lasted one class period for each class. For copying, the text titled “Famous Painter” was projected onto the smart board, and students were asked to write the text in their notebooks. On the second day of the data collection process, the text titled “The Shoe Repairman” was read aloud to the students by the researcher, and writing data from dictation was collected in this manner. Finally, to assess their free writing skills, students were asked to write about their dream career. Thus, the data collection process spanned three workdays, during which data was collected across three dimensions. Consequently, the data collection process began on 6 April 2026 and continued for three working days, ending on 8 April 2026.

The handwriting of fourth-grade students in three different areas (copying, dictation, and free writing) was scanned and digitized. The resulting student handwriting samples were analyzed for legibility by the researcher (Halil İbrahim Öksüz) using ChatGPT. The AI-based evaluation process was conducted between April 15 and April 20, 2026, utilizing the ChatGPT-4o model. To ensure high-quality visual input for the analysis, all handwriting samples were scanned at a resolution of 300 dpi and uploaded to the system in JPG format. To preserve the independence of the evaluations and prevent any sequential bias that might arise in the AI system, each of the 201 handwriting samples was processed as a separate task. In addition, the AI model was instructed to evaluate each text without referring to previous samples. First, ChatGPT was provided with the Multidimensional Legibility Scale and necessary introductory information about the study. Subsequently, specific instructions were given, and ChatGPT was asked to analyze a handwriting sample not used in the current study according to the Multidimensional Legibility Scale. Since the score assigned by ChatGPT to the relevant handwriting matched the score given by the researcher, the process of evaluating the fourth-grade students’ handwriting was initiated. The same handwriting samples were also scored by another researcher (İsmail Yaşartürk) according to the Multidimensional Legibility Scale. To ensure the researcher was not influenced by the AI, two separate scoring processes were conducted by two researchers, and the data collection process was completed.

The prompts given to ChatGPT are as follows:

You are a researcher specializing in Turkish language education who focuses on handwriting legibility. Your task is to objectively and scientifically analyze handwriting samples from fourth-grade elementary school students. Using the Multidimensional Legibility Scale described below, you will score the student handwriting samples provided to you based on the dimensions of slant, spacing, form, and line tracking. The Multidimensional Legibility Scale is as follows.

- *Slant*
  1. *point: Letters are quite irregular. The writing exhibits both right and left slant (mixed).*
  2. *points: Although the letters do not conform to exact measurements, the writing is slanted to the right or left, and this slant continues partially throughout the text.*
  3. *points: The letters are written neatly and completely at a 90-degree angle throughout the text.*
- *Spacing*
  1. *point: Spacing between letters, words, and sentences is inconsistent and insufficient throughout the text.*
  2. *points: There are some inconsistencies in the spacing between letters, words, and sentences. Spacing is not consistent throughout the text.*
  3. *points: Spacing between letters, words, and sentences is appropriate and consistent throughout the text.*
- *Size*
  1. *point: The letters are too small to read and irregular in size. The ratio of uppercase to lowercase letters is also unbalanced and inconsistent.*
  2. *points: Although the letters are larger or smaller than normal, their size is consistent. There are also imbalances in the ratio of uppercase to lowercase letters.*
  3. *points: The letters are of a readable size, and this size is consistent throughout the text. The uppercase-to-lowercase ratio is entirely appropriate.*
- *Form*
  1. *point: The letters are written irregularly, and the starting and ending points of the ascenders and descenders are inadequate.*
  2. *points: There are some deficiencies in the starting and ending points of the letters, as well as in the proportions of the ascenders and descenders.*
  3. *points: The letters are written in full compliance with the rules. Start and end points are properly and correctly executed. Ascenders and descenders are proportionate.*
- *Line Tracking*
  1. *point: Line tracking is quite inadequate. Constant slippage downward or upward occurs. Line-end overhangs and spacing irregularities are excessive.*
  2. *points: Occasional slippage downward or upward occurs in line tracking. Line-end overflows and spacing irregularities are visible, albeit to a lesser extent.*
  3. *points: Line spacing is quite consistent. There is no text extending above or below the line. Spacing at the end of lines is consistent, and there are no overflows.*

*Rules to follow: Evaluate each text independently, remain objective, and justify the score you assign for each sub-criterion.*

ChatGPT evaluations were conducted using a standardized set of prompts based on the Multidimensional Legibility Scale. All dimensions included in the scale (slant, spacing, size, form, and line tracking) were explicitly presented to the model, and the evaluations were instructed to be based solely on the characteristics reflected in the text. Additionally, an academic persona was assigned to ChatGPT to standardize its decisions.

### 2.4. Data analysis

Data collected from fourth-grade elementary school students was analyzed using the SPSS 24 software package (IBM Corp., 2016). In the study, skewness and kurtosis values were first examined to determine whether the data met the assumption of normality. The results of the analysis indicated that the data followed a normal distribution. This is because all variables in the study fell within the ± 2 range in terms of skewness and kurtosis. For this reason, it was assumed that the data followed a normal distribution [32]. The assessments were derived from two independent scorers (a human assessor and an artificial intelligence modelling system). The aim here is not to compare individual paper-based scoring discrepancies, but rather to compare the averages of the overall score distributions produced by these two systems. In other words, the focus of the present study is not on the writing skills of the students from whom the writing samples were taken, but on comparing the assessors. For this reason, and given that the data followed a normal distribution, an independent samples t-test was used, whilst a Pearson correlation test was employed to assess the level of correlation between the two assessors.

### 2.5. Ethics statement

Prior to data collection for this study, the necessary permissions for the research were obtained from the Scientific Research and Publication Ethics Committee of Tokat Gaziosmanpaşa University at its meeting No. 03 held on 26 March 2026 (Decision No. 706861 dated 5 March 2026 – application by Ramazan DENİZ). All procedures were carried out in accordance with the ethical standards of the Declaration of Helsinki.

## 3. Results

This section presents descriptive statistics for the study, normality statistics for the variables, and tests conducted to compare two different raters (ChatGPT versus the researcher).

The students’ writings—produced by looking at a text, dictation, and free writing—were scored by the researcher (İsmail Yaşartürk), and the resulting data are presented in Table 1. According to the table, it was determined that the data follow a normal distribution, as the skewness and kurtosis values fall within the ± 2 range.

**Table 1:** Descriptive statistics and normality levels for variables rated by the researcher.

Variables	n	x̄	SD	Skewness	Kurtosis
Copying	67	11.34	1.92	−.323	−.804
Dictation	67	12.28	1.82	−.234	−.883
Free Writing	67	12.36	1.64	.121	−.277

The students’ written work—produced through copying, dictation, and free writing—was scored by ChatGPT, and the resulting data are presented in Table 2. According to the table, the data were found to follow a normal distribution, as the skewness and kurtosis values fell within the ± 2 range. Based on the normality data obtained, an Independent Samples t-Test was applied to compare the writing skills of students graded by two different graders, and the results are presented in Tables 3–5.

**Table 2:** Descriptive statistics and normality levels for variables scored by ChatGPT.

Variables	n	x̄	SD	Skewness	Kurtosis
Copying	67	11.79	1.93	−.406	−.594
Dictation	67	12.46	1.84	−.082	−.925
Free Writing	67	12.51	1.77	−.224	−.541

**Table 3:** Comparison of writing scores given by the researcher and ChatGPT for copying.

	Group	n	x̄	SS	df	t	*p*
Slant	Researcher	67	2.31	.56	66	−1.903	.060
	ChatGPT	67	2.50	.53			
Spacing	Researcher	67	2.04	.47	66	−1.448	.150
	ChatGPT	67	2.16	.48			
Size	Researcher	67	2.27	.62	66	.643	.522
	ChatGPT	67	2.19	.72			
Form	Researcher	67	2.07	.66	66	−1.459	.147
	ChatGPT	67	2.22	.52			
Line Tracking	Researcher	67	2.67	.53	66	−.336	.66
	ChatGPT	67	2.70	.49			
Total Copying	Researcher	67	11.35	1.92	66	−1.345	.181
	ChatGPT	67	11.79	1.93			

**Table 4:** Comparison of dictation scores provided by the researcher and ChatGPT.

	Group	n	x̄	SD	df	t	*p*
Slant	Researcher	67	2.37	.57	66	−1.926	.056
	ChatGPT	67	2.56	.50			
Spacing	Researcher	67	2.27	.51	66	−.824	.413
	ChatGPT	67	2.25	.45			
Size	Researcher	67	2.42	.63	66	.439	.661
	ChatGPT	67	2.37	.55			
Form	Researcher	67	2.40	.58	66	.606	.546
	ChatGPT	67	2.42	.52			
Line Tracking	Researcher	67	2.78	.45	66	−.590	.556
	ChatGPT	67	2.82	.42			
Total Dictation	Researcher	67	12.28	1.81	66	−.568	.571
	ChatGPT	67	12.46	1.84			

**Table 5:** Comparison of free writing scores provided by the researcher and ChatGPT.

	Group	n	x̄	SD	df	t	*p*
Slant	Researcher	67	2.31	.47	66	−2.541	.013*
	ChatGPT	67	2.54	.56			
Spacing	Researcher	67	2.43	.50	66	.523	.606
	ChatGPT	67	2.39	.59			
Size	Researcher	67	2.52	.56	66	.299	.765
	ChatGPT	67	2.50	.51			
Form	Researcher	67	2.36	.45	66	1.603	.111
	ChatGPT	67	2.22	.48			
Line Tracking	Researcher	67	2.73	.48	66	−1.353	.178
	ChatGPT	67	2.84	.41			
Total Free Writing	Researcher	67	12.36	1.64	66	−.506	.613
	ChatGPT	67	12.51	1.77			

*p < .05.

The findings regarding the comparison of scores obtained from the evaluation of students’ copying skills by the researcher and ChatGPT, based on the Multidimensional Legibility Scale, in terms of subscales and total scores are presented in Table 3. According to the paired samples t-test results, no significant differences were detected between the researcher’s and ChatGPT’s evaluations in terms of slant (t(66)=−1.903, p > .05), spacing (t(66)=−1.448, p > .05), size (t(66)=.643, p > . 05), form (t(66)=−1.459, p > .05), line tracking (t(66)=−.336, p > .05), and total score (t(66)=−1.345, p > .05). Based on these results, it can be concluded that ChatGPT is a reliable evaluator for assessing the legibility of handwritten texts produced by fourth-grade elementary school students.

The findings regarding the comparison of scores obtained from the evaluation of students’ dictation skills by the researcher and ChatGPT, based on the Multidimensional Legibility Scale, in terms of subscales and total scores are presented in Table 4. According to the paired-samples t-test results, no significant differences were detected between the researcher’s and ChatGPT’s evaluations in terms of slant (t(66)=−1.926, p > .05), spacing (t(66)=−.824, p > .05), size (t(66)=.439, p > . 05), form (t(66) = .546, p > .05), line tracking (t(66) = −.590, p > .05), and total score (t(66) = −.568, p > .05). Based on these results, it can be concluded that ChatGPT is a reliable evaluator for assessing the legibility of dictation-based writing by fourth-grade elementary school students.

The findings regarding the comparison of scores obtained from the evaluation of students’ free-writing skills by the researcher and ChatGPT, based on the Multidimensional Legibility Scale, in terms of subscales and total scores are presented in Table 5. According to the table, there is a significant difference between the researcher’s and ChatGPT’s evaluations regarding the slant (t(66) = −2.541, p < .05). This difference favors the readability assessment performed by ChatGPT. Again, according to the table, no significant differences were detected regarding spacing (t(66) = .523, p > .05), size (t(66) = .299, p > .05), form (t(66) = 1.603, p > .05), line tracking (t(66) = −1.353, p > . 05), and total score (t(66) = −.506, p > .05). Based on these results, it can be concluded that ChatGPT is generally a reliable evaluator of the legibility of handwritten texts produced by fourth-grade elementary school students.

Upon examining Table 6, the essays scored by the researcher and those scored by ChatGPT showed a positive correlation in both copying (r(67) = .78, p = .000), dictation (r(67) = .71, p = .000), and free writing (r(67) = .80, p = .000). These results also indicate that the readability assessments conducted by the researcher and ChatGPT show a high degree of agreement. Furthermore, To examine the inter-rater agreement between the human researcher and ChatGPT, an Intraclass Correlation Coefficient (ICC) was calculated using a two-way mixed-effects model with an absolute agreement definition. The single-measures ICC was determined to be .763 (95% CI [.695, .817], F(199, 199) = 7.667, p < .001). This result indicates a good level of inter-rater reliability between the human evaluator and artificial intelligence.

**Table 6:** Results of the Pearson Correlation Test between raters.

	Researcher Copying	ChatGPT Copying
Researcher Copying	1	–
ChatGPT Copying	.78**	1
	Researcher Dictation	ChatGPT Dictation
Researcher Dictation	1	–
ChatGPT Dictation	.71**	1
	Researcher Free Writing	ChatGPT Free Writing
Researcher Free Writing	1	–
ChatGPT Free Writing	.80**	1

Furthermore, using the prompts shared by the researcher under the heading ‘Data Collection’, the same student writings were re-evaluated one month later by another researcher (İsmail Yaşartürk). This process was carried out to assess the consistency of ChatGPT’s evaluations. Accordingly, to assess the evaluator reliability (test-retest stability) of the AI model, a subset of handwriting samples (N = 67) was re-evaluated by ChatGPT one month later using the same prompts. An Intraclass Correlation Coefficient (ICC) analysis based on a two-way mixed-effects model was carried out, using absolute agreement as the basis. The single-measure ICC value was determined to be 0.922 (95% CI [0.877; 0.952], F(66, 66) = 24.432, p < 0.001); this result indicates an excellent level of intra-rater reliability and high temporal stability in AI scoring.

## 4. Discussion

The study’s main findings revealed a strong, positive, and statistically significant correlation between artificial intelligence (ChatGPT) and human evaluators. These results are consistent with numerous recent studies showing that ChatGPT can perform similarly to human evaluators when assessing written text [33–35]. In particular, the absence of a statistically significant difference in the total scores for dictation, copying, and free-writing tasks indicates that artificial intelligence is capable of providing consistent evaluations across different types of writing [35,36]. The literature frequently notes that the GPT-4 model, in particular, shows a very high degree of similarity with human evaluators in assessing surface-level structural elements such as grammar, vocabulary, and mechanical features [34,37,38]. It is thought that this strong correlation identified in the study can be explained by the fact that artificial intelligence is not subject to human factors such as loss of objectivity or fatigue. While human evaluators may be influenced by factors such as fatigue, subjectivity, mood swings, or bias, systems like ChatGPT are able to apply predefined criteria to every text with the same consistency [20,36,39]. This represents a multifaceted advantage that could alleviate the assessment burden on teachers—particularly in classes with large numbers of students—and enhance transparency in the grading process [40–42]. In contrast, one of the most striking findings of the study is the significant difference in favor of ChatGPT in the “slant” sub-dimension of the free writing task. This can be explained by a fundamental technical divergence in how artificial intelligence processes text. While human evaluators typically employ an intuitive and holistic approach—often referred to as “subjective visual assessment”—during the evaluation process, ChatGPT analyzes the data with algorithmic precision [20,33].

From a technical perspective, while the human eye evaluates geometric details such as text slant or alignment, it may be influenced by psychological factors such as fatigue, text density, or subjectivity [36]. In contrast, by operating with precision as a visual-linguistic evaluation tool based on evaluation criteria, it identifies the structural features of the text through advanced multimodal processing [38,43]. This issue is explained in the literature through the concepts of “proxy” and “trins” [43,44]. While human evaluators focus on contextual, creative, and intentional variables that convey deeper meaning, AI tools score text based on numerical proxy variables such as word count, sentence length, and structural arrangement [44,45]. ChatGPT’s tendency to assign higher scores in this sub-dimension may stem from its ability to perceive the syntactic features of the text with an overly standardized and robotic precision—beyond what the human eye can detect—and to algorithmically reward this structural consistency [36,38]. Therefore, artificial intelligence’s sensitivity in this sub-dimension may actually stem from its ability to process structural arrangements and complex data through systematic, rating-scale-based visual evaluation—a process independent of human emotions and fatigue [35,40].

Similarly, the literature indicates that AI tools tend to evaluate texts in a more quantitative and objective manner, whereas human evaluators are influenced by the qualitative aspects of writing, such as context, intent, or creativity [43,44]. It is also noted that ChatGPT may capture certain structural parameters of a text—such as trends or flow patterns—with a mathematical precision that the human eye cannot detect, particularly in cognitively demanding processes like free writing [38,46]. Nevertheless, ChatGPT’s focus solely on structural features also highlights its limitations regarding contextual understanding and deep semantic coherence. Many studies also argue that artificial intelligence is not as competent as human experts in identifying creativity, rhetorical nuances, and off-topic content in text [33,41,47]. In particular, studies indicate that a lenient scoring tendency can lead AI to assign higher scores to even low-quality texts solely due to structural correctness [33,36]. Based on this, it is argued that rather than serving as an evaluator on its own, artificial intelligence should be supported by a well-structured rubric and guidelines [38,48,49]. In conclusion, the findings of this study support the view that a hybrid model based on human-AI collaboration is the most effective approach, contrary to the notion that artificial intelligence will completely replace humans in writing instruction [42,50,51]. While AI boosts student motivation by quickly identifying technical and physical errors and providing immediate feedback [52], Teachers must have the final say regarding the deeper meaning and contextual appropriateness of a written text as assessed by students [20,41].

## 5. Conclusions

This study, which aimed to provide evidence regarding the potential of artificial intelligence systems to support teachers in the writing assessment process by examining the level of score agreement and assessment accuracy between ChatGPT and human raters, demonstrated that ChatGPT produces highly consistent and reliable results when evaluating elementary students’ performance on copying, dictation, and free-writing tasks compared to human raters. The data obtained confirm the consistent results reported in the literature, showing no statistically significant difference in total scores between the two raters and a strong, positive correlation ranging from 0.71 to 0.80 [34–36]. This clearly demonstrates that generative AI tools have the potential to serve as an objective, consistent, and efficient assessment aid for educators. The speed and consistency of AI in technical and structural dimensions offer significant opportunities to alleviate the heavy assessment burden on teachers. However, the significant difference observed in the free-writing task supports the idea that AI’s approach to text relies more on superficial structural variables, whereas human evaluators focus on deep semantic context [33,44].

Furthermore, informal observations made during the research process indicate that, despite ChatGPT’s limitations in terms of semantic consistency, creativity, argumentative strength, and the ability to distinguish off-topic content, the contextual sensitivity inherent in human judgment will remain indispensable in the evaluation process. In conclusion, this study supports the view of a hybrid model that combines the strengths of both parties, rather than artificial intelligence completely replacing humans in educational processes [50,51]. While the immediate and personalized feedback provided by artificial intelligence enhances student motivation and autonomy, teachers’ pedagogical expertise and interpretive depth will continue to safeguard the validity of the assessment process. In future educational settings, this balanced model—which combines the speed of artificial intelligence with the pedagogical depth of humans—will serve as a crucial tool for sustainable success.

### 5.1. Limitations

The ability to provide instant and personalized feedback offered by generative AI has the potential to enhance students’ motivation and autonomy in writing. While delays in feedback in traditional methods slow down the learning cycle, the real-time analysis provided by tools like ChatGPT can enable students to identify and correct their mistakes immediately. However, a strategic limitation in this process is the need to develop the skills of both teachers and students in providing clear instructions to ChatGPT, ensuring that the AI focuses not only on technical errors but also on semantic coherence.

Although the findings of this study highlight the potential of artificial intelligence in assessment processes, there are a number of important limitations that must be considered when interpreting the results. First, the limited sample size of the study and the fact that it included only fourth-grade elementary school students make it difficult to generalize the findings to different educational levels or broader student populations. The writing patterns of students at different proficiency levels may affect the consistency of AI scoring in various ways.

Another limitation relates to the nature of the AI technology used. This study was conducted using a specific version of ChatGPT (GPT-4o). However, large language models are updated at a dizzying pace, and each new version introduces significant changes in scoring capabilities. Therefore, the findings of this research represent a specific technological moment, and it should be noted that scoring accuracy may change with future updates.

Another limitation is that, in the process of integrating artificial intelligence technologies into educational settings, standards for appropriate use and academic integrity must be clearly defined. For artificial intelligence to serve as a reliable grader, the rubrics and system prompts used must be structured in a transparent, unbiased, and student-appropriate manner, which is critical for ensuring educational equity. In this context, incorporating artificial intelligence literacy training into the curriculum will foster the development of conscious and responsible users of the technology.

### 5.2. Future research

Based on the findings of this study, a series of recommendations can be made for future research and educational settings. First, there is a need for more comprehensive studies examining how AI’s grading performance varies across different proficiency levels. In particular, including participants from different educational levels will strengthen the generalizability of the findings.

Second, longitudinal studies can be planned to track the long-term effects of AI-powered assessment tools on student development. This is because, when used correctly, AI serves as a reward that facilitates student progress, but when misused, it can lead to cognitive decline.

As a third area of research, potential bias and fairness issues in AI-driven grading processes should be investigated across different demographic groups. Additionally, developing hybrid models that maximize AI’s capacity to serve not merely as a technical grader but as an instructional feedback partner is crucial for future educational environments.
